# Proximity to crop relatives determines some patterns of natural selection in a wild sunflower

**DOI:** 10.1111/eva.13201

**Published:** 2021-03-12

**Authors:** Nora Mitchell, Scott A. Chamberlain, Kenneth D. Whitney

**Affiliations:** ^1^ Department of Biology University of Wisconsin – Eau Claire Eau Claire WI USA; ^2^ Department of Ecology & Evolutionary Biology Rice University Houston TX USA; ^3^ Department of Biology University of New Mexico Albuquerque NM USA; ^4^ Present address: rOpenSci Department of Environmental Science, Policy and Management University of California Berkeley CA USA

**Keywords:** antagonist, geographic mosaic, herbivory, mutualist, natural selection, phenotypic selection analysis, pollinators, seed predators

## Abstract

Abiotic and biotic heterogeneity result in divergent patterns of natural selection in nature, with important consequences for fundamental evolutionary processes including local adaptation, speciation, and diversification. However, increasing amounts of the global terrestrial surface are homogenized by agriculture (which covers nearly 50% of terrestrial vegetated land surface) and other anthropogenic activities. Agricultural intensification leads to highly simplified biotic communities for many taxa, which may alter natural selection through biotic selective agents. In particular, the presence of crops may alter selection on traits of closely related wild relatives via shared mutualists and antagonists such as pollinators and herbivores. We asked how the presence of crop sunflowers (*Helianthus annuus*) alters natural selection on reproductive traits of wild sunflowers (*Helianthus annuus texanus*). Across two years and multiple sites, we planted replicated paired populations of wild *H. a. texanus* bordering sunflower crop fields versus approximately 2.5 km away. We measured fitness, floral traits, and interactions of the plants with insect pollinators and seed predators. We found limited evidence that proximity to crop sunflowers altered selection on individual traits, as total or direct selection differed by proximity for only three of eleven traits: ray length (a marginally significant effect), *Isophrictis* (Gelechiidae, moth) attack, and *Neolasioptera* (Cecidomyiidae, midge) attack. Direct (but not total) selection was significantly more heterogenous far from crop sunflowers relative to near crop sunflowers. Both mutualist pollinators and antagonist seed predators mediated differences in selection in some population‐pairs near versus far from crop sunflowers. Here, we demonstrate that agriculture can influence the evolution of wild species via altered selection arising from shared biotic interactions, complementing previously demonstrated evolutionary effects via hybridization.

## 1 INTRODUCTION

In natural landscapes, abiotic and biotic heterogeneity produce spatially divergent patterns of natural selection, contributing to divergent evolutionary paths among populations and influencing longer‐term processes such as local adaptation, speciation, and evolutionary diversification. However, reduction in this natural heterogeneity via anthropogenic alterations such as urbanization, agriculture, and introduction of invasive species could reduce natural geographic variation in evolutionary trajectories (Lau, 2006; Palkovacs et al., 2012). Despite the fact that croplands, pastures, and rangelands covered ~50% of the global vegetated land surface as of 2005 (Foley et al., 2005), we lack a thorough understanding of how agriculture alters the evolution of co‐occurring wild plants through natural selection.

Proximity to agriculture may lead to altered evolutionary trajectories for wild species in various ways. First, drastically increased use of herbicides associated with genetically modified crops (GMO; e.g., Roundup) has led to evolution of resistance to the herbicide in many wild species (reviewed in Délye et al., 2013). Second, fertilizer runoff from crop fields affects growth and other responses of plants along crop borders (Blackshaw et al., 2004; Quinn et al., 2007), which could drive evolution of resource‐acquisition traits and competitive ability. Third, changes in composition in, and homogenization of, biotic communities associated with agriculture (Chamberlain et al., 2013; Ekroos et al., 2010; Gámez‐Virués et al., 2015) may affect evolution by natural selection in wild species via alteration of the abundance or behavior of selective agents. Given that many wild species now occur in human‐altered landscapes, it is likely that their evolution is affected by anthropogenic homogenization. Although there are a few cases documenting concurrent cases of homogenization across taxonomic groups (i.e., Carvalheiro et al., 2013), we know little of how biotic homogenization influences evolution in wild species. To our knowledge, no studies have experimentally examined the possible evolutionary consequences of landscape‐level homogenization of biotic interactions, which requires experiments in multiple populations and a geographic perspective.

In addition to the effects detailed above, the ability of a crop to influence evolution in nearby wild plants may depend on their degree of relatedness. Crop‐to‐wild gene flow commonly occurs and could affect the evolution of wild species (Ellstrand et al., 1999; Pilson & Prendeville, 2004), and patterns of natural selection could also be altered if species are closely related. We know that species interactions are often phylogenetically conserved, such that closely related species are likely to interact with similar species, or at least have a similar number of interactions (Gómez et al., 2010). Thus, if the crop and focal wild plant species are closely related, they may interact with many of the same species (e.g., share pollinators and herbivores) and furthermore may respond similarly to biotic and abiotic conditions because traits are often phylogenetically conserved (Blomberg et al., 2003). However, since crops have been artificially selected to be morphologically and phenologically distinct from their wild relatives, we note that traits may not always be phylogenetically conserved between crops and their wild relatives. Interactions between crops and wild relatives are especially likely when they occur in close proximity. Such situations are likely quite common; many crop plants are cultivated in locations where their wild relatives are abundant and diverse. Examples include sunflowers in North America, wheat in the Middle East, corn, squash, and peppers in Mexico, and potatoes from southwestern USA to Uruguay (Jarvis et al., 2008).

Cultivated *Helianthus annuus* and its wild congeners (sunflowers; Asteraceae) provide a highly tractable system for studying how agriculture alters the evolutionary trajectories of wild species in situations where crops and wild species occur in close proximity and may therefore share mutualists and antagonists. First, there is both temporal and spatial overlap between crop and wild sunflowers in sunflower‐growing regions of the US. Crop and wild sunflowers can overlap for 5–6 months in flowering phenology (K. Whitney, personal observation), and wild *Helianthus* commonly occur along the borders of sunflower crop fields (Burke et al., 2002). Second, this overlap provides high potential for shared pollinators (mutualists) and seed predators (antagonists) among crop and wild sunflowers. A diverse biotic community interacts with wild and crop sunflowers. Across their ranges, the pollinator communities of both crop and wild sunflowers are dominated by several hundred species of bees, some of which are shared between *Helianthus* species (Hurd et al., 1980), with honeybees particularly prevalent in crop sunflowers (Greenleaf & Kremen, 2006). Within a more restricted region, our research group has observed 32 pollinator species (23 of which are bee species) (Chamberlain et al., 2013). Crop sunflowers have been artificially selected for larger inflorescences (flower heads) (Seiler, 1997), and therefore, floral traits of wild species in particular may be subject to differences in selection due to proximity to showy crop inflorescences. Many species of insect seed predators attack both wild and crop sunflowers (Charlet et al., 1997), and their species‐specific damage to sunflower seeds is easily quantified. Resistance to these seed predators is mediated by both physical and chemical defenses, such as sesquiterpene lactones (Rogers et al., 1987) and is under natural selection (Whitney et al., 2006).

Here, we explore how proximity of crop sunflowers (*Helianthus annuus* L.) to a wild North American sunflower (*Helianthus annuus* ssp. *texanus* Heiser) alters natural selection on floral traits and resistance to insect seed damage in the latter. Specifically, we ask the following three questions: (1) How does proximity to crop sunflowers affect total and direct selection on *H. a. texanus* floral traits?; (2) Across replicate populations, does homogeneity of selection differentials and selection gradients for *H. a. texanus* floral traits differ with proximity to crop sunflowers?; and (3) Does selection mediated by mutualists and antagonists differ with proximity to crop sunflowers?

## 2 MATERIALS AND METHODS

### 2.1 Experimental design

**FIGURE 1:**
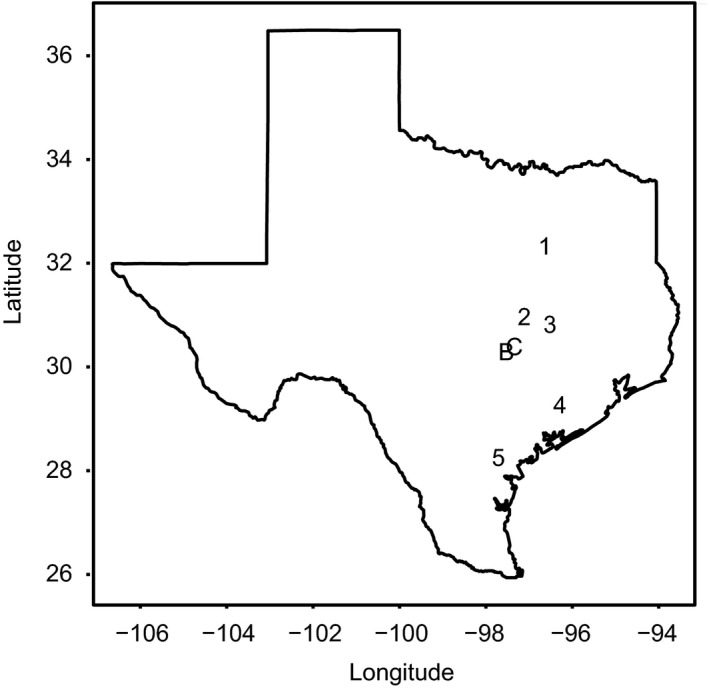
Map of natural populations from which seeds were collected in 2009 (populations B and C) and where experimental studies were conducted in 2010 and 2011 (Sites 1–5). Note that five sites (1–5) were used in 2010, of which two sites (1 and 2) were also used in 2011

In experimental studies in 2010, we manipulated the proximity of *H. a. texanus* to crop sunflowers by transplanting arrays of 80–155 seedlings (hereafter, “populations”) either near crop sunflowers (plot of *H. a. texanus* 10 m from the crop) or far from them (plot 2.5 km from any sunflower crop). The “far” populations were adjacent to semi‐natural habitats (e.g., tree lines, forest patches) which themselves bordered either fallow fields or other non‐sunflower crops (e.g., sorghum, cotton, corn, rice, or sesame). We replicated near and far populations (“population‐pairs”) from two seed sources (B and C, collected in 2009, Figure 1) at five farms (“sites”) in Texas (Figure 1) for a total of 20 individual plots to enhance the generality of results. These sites were all well within the natural range of wild *H. a. texanus*, and wild populations were often seen near to our sites (S. Chamberlain, personal observation. All the sunflower crops in this study were grown for oil production and were planted according to each farm's standard practices (i.e., the crop planting was not influenced by the investigators). The crop sunflowers were all Clearfield**^®^** variety, which are not genetically modified, but have been artificially selected to be resistant to the imidazolinone herbicides (Sala et al., 2008), which were sprayed on the crop sunflowers to reduce weeds. In 2011, we used the same design as in 2010 (proximity treatment crossed with seed origin treatment), but only used two of the five sites used in 2010 (Sites 1 and 2; see Figure 1). In 2010, we lost one population at Site 1 due to accidental herbicide spraying. At Site 4, we lost one far population to flooding, and the other populations at Site 4 experienced high early mortality resulting in low sample sizes for traits, so we omitted Site 4 from further analysis. In 2011, an extreme drought caused wild pigs to seek out wet roots early in the season and damage plants in two populations (one near, one far) at Site 2; we replaced these plants with new seedlings.

We obtained seedlings by nicking seeds with a razor blade and germinating them on damp filter paper in late February each year (2010 and 2011). We kept germinating seeds in the absence of light at room temperature and moved them into the light after they produced fine root hairs. We kept seeds damp at all times during germination. We transplanted approximately eight‐day‐old seedlings into peat pellets (J30100 Super; Jiffy, Denmark) and grew them in a Rice University greenhouse for approximately 4 weeks before transplanting to the field in approximately early‐ to mid‐April, to match the rough size and phenology of wild *H. a. texanus* individuals in this region. To aid establishment, we watered plants in the field every three to five days by hand for approximately 10 days.

### 2.2 Fitness measures

We quantified fitness as whole plant seed production for each plant. We used mesh bags (8 cm × 8 cm, made from plastic mesh; DelStar Technologies) to capture seeds from three to six inflorescences per plant when possible, chosen haphazardly (Whitney et al., 2006). In September, after seeds had matured and plants had senesced, we counted the total number of inflorescences per plant (range 0–310) and collected bagged inflorescences. Mean seed production per inflorescence was counted and multiplied by inflorescence number to estimate whole plant seed production. *H. a. annuus* is an annual, so this is a measure of lifetime fitness. To account for possible scaling of seed production and flower traits with plant size, at the end of the season we measured height to the tallest inflorescence (to the nearest cm) and diameter of the stem at the base (to the nearest 0.1 mm) (Whitney et al., 2006). We calculated plant stem volume as $\pi r^{2}h$, where *r* is the radius of the stem at the base, and *h* is the height.

### 2.3 Inflorescence and floral trait measurements

**FIGURE 2:**
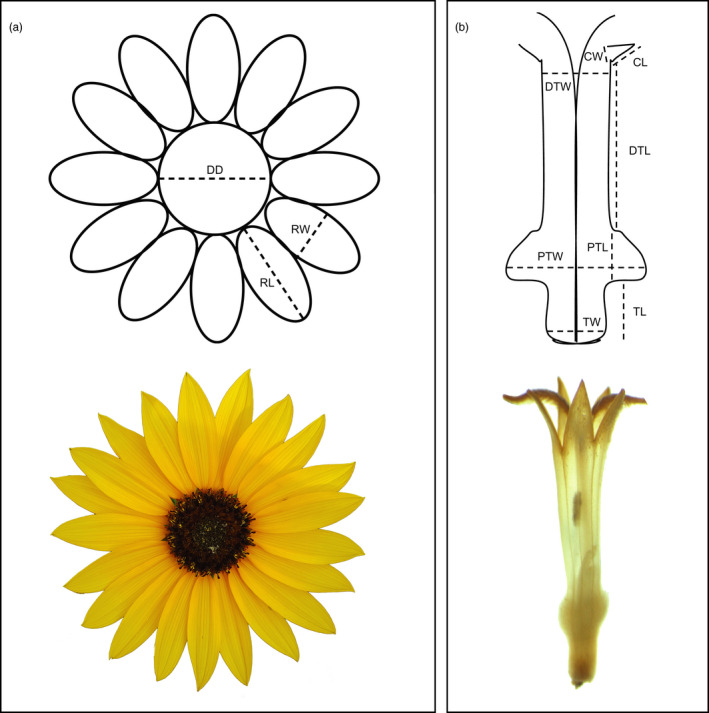
Diagram representing traits measured on (a) inflorescences and (b) individual disk flowers in *H. a. texanus*. Radiate inflorescences in the family Asteraceae consist of non‐reproductive marginal ray florets and bisexual central disk florets (up to 300 disk florets per head in *H. a. texanus*). CL, corolla lobe length; CW, corolla lobe width; DD, disk diameter; DTL, distal throat length; DTW, distal throat width; PTL, proximal throat length; PTW, proximal throat width; RL, ray length; RW, ray width; TL, corolla tube length; TW, corolla tube width. The following traits were calculated using multiple traits: CS (corolla size = CL × CW), proximal throat size (PTS = PTL × PTW), and corolla tube size (TS = TL × TW). Note the disk flower has been stored in alcohol and thus has lost some of its normal yellow and brown pigmentation.Photographs by N. Mitchell and S. Chamberlain

*Helianthus* inflorescences consist of non‐reproductive marginal ray florets and bisexual central disk florets (Seiler, 1997). We measured nine floral traits on each plant: four on the scale of inflorescences (disk diameter DD, ray length RL, ray width RW, number of rays NR, to the nearest 0.01 mm (see Figure 2a), and five on the scale of individual disk flowers (as explained below). In doing so, we took a broad approach to quantifying inflorescence and floral morphology (examining every major dimension) to reduce bias associated with focusing on dimensions that we a priori believed likely to be under selection. We do, however, note that many of these traits are biologically relevant, for instance ray length (RL), number of rays (NR), and disk diameter (DD) are the major “display” traits used to attract pollinators and may therefore be under selection. Similarly, aspects of disk floret size (such as the size of the corolla tube) might be expected to be under selection as this trait is classically known to limit access to some pollinators, while allowing access by others (reviewed in Harder & Johnson, 2009).

We collected up to five individual disk flowers in 70% ethanol from different inflorescences on each plant across the flowering season; we then averaged measurements across the disk flowers to obtain a single value per trait per plant. We captured pictures of each individual disk flower using a Leica DFC‐480 digital camera attached to a Leica DM‐2500 dissecting microscope camera and Leica Application Suite (Leica Microsystems) and then took eight measurements (corolla lobe length, corolla lobe width, distal throat width, distal throat length, proximal throat length, proximal throat width, corolla tube length, corolla tube width; see Figure 2b) using Image J software (Rasband, 1997). Using these eight measurements, we then calculated values for five traits for each individual disk flower as: corolla lobe size CS (corolla lobe length × width), distal throat width (as is), distal throat length DTL (as is), proximal throat size PTS (proximal throat length × width), and corolla tube size TS (corolla tube length × width). Five of these inflorescence and floral traits had estimated narrow‐sense heritability estimates that differed from zero and four traits that did not (Appendix S1, Table S1).

### 2.4 Seed predator damage

We estimated seed predator damage (by antagonists) on all *H. a. texanus* plants in each population by capturing and examining seeds from three to six inflorescences per plant. We placed a mesh bag on each inflorescence after pollination, but before seed drop occurred, allowing ample time for seed predators to interact with the inflorescence. We collected bagged inflorescences at the end of the season, after seeds had matured and plants had senesced. We pooled all inflorescences, and then sub‐sampled ca. 80 seeds with an ×10 dissecting microscope to quantify taxon‐specific damage inflicted by the sunflower midge *Neolasioptera helianthi* (Diptera: Cecidomyiidae) and hole damage by the moth genus *Isophrictis* (Lepidoptera: Gelechiidae). Attack by these seed predators results in destruction of the seed. We then calculated damage for each species as a proportion (number of damaged seeds/total number of seeds examined).

### 2.5 Pollen deposition

We used pollen deposition as an estimate of pollinator visitation rate on the individual plant level (Cayenne Engel & Irwin, 2003) allowing us to connect pollinator behavior to selection on floral traits. Because this trait is time‐consuming to measure, we focused on two focal sites (Sites 1 and 2) in both 2010 and 2011 with one far population and one near population (a population‐pair) for a single seed source measured at each. We collected stigmas (mean = 5.9 stigmas per plant, range = 1–21) in the field from up to eight inflorescences per plant during the season. We pressed stigmas under a microscope slide in glycerin, photographed them with fluorescence microscopy, and counted pollen grains with a macro program written by SAC for Image J (Rasband, 1997). We estimated pollen deposition per plant (average no. pollen grains/flower × 100 flowers/inflorescence × no. inflorescences). We assume a constant number of flowers per inflorescence (100) as we do not have data on variation in this trait. There was no evidence of pollen limitation either near or far from sunflower crops (Appendix S2).

### 2.6 Phenotypic selection analyses

For each population, we performed phenotypic selection analysis (PSA) following Lande and Arnold (1983). PSA is a statistical method to detect natural selection on phenotypic traits within a generation and does not quantify trait change between generations. Selection differentials (*s*') represent total selection on a trait, the combination of direct selection on the trait plus indirect selection arising from selection on correlated traits. Selection gradients (*β*) represent direct selection on each trait after indirect selection has been removed. We used log‐transformed relative fitness (calculated as seed production of an individual divided by the population mean seed production) in the analyses. We estimated selection on four inflorescence traits (disk diameter, DD; ray length, RL; ray width, RW; number of rays, NR), five disk floral traits (corolla lobe size, CS; distal throat width, DTW; distal throat length, DTL; proximal throat size, PTS; corolla tube size, TS), and two antagonist traits (*Isophrictis* attack, ISO; *Neolasioptera* attack, NEO). We acknowledge that environment‐fitness covariance could lead to non‐independence of fitness and some of these traits (Rausher, 1992), which could be circumvented using a genotypic selection analysis, but this type of analysis was not possible given our seed stocks. See Table S2 for sample sizes for each trait and population included in these analyses. We also included plant stem volume in the multiple regression to account for indirect selection on floral traits via direct selection on plant size. We transformed all traits as necessary to improve normality and then standardized them within populations (mean = 0, SD = 1). We checked diagnostics for normality of residuals and violations of multicollinearity and excluded one population from selection gradient analysis and downstream analyses (Site 1, seed source C, near population) for violating these assumptions. For the remaining populations (*n* = 22), variance inflation factors (VIFs) were <9.7 and all condition indices were <11.3, falling below the thresholds of 10 and 30, respectively, thus suggesting that multicollinearity is unlikely to compromise the results (Myers & Myers, 1990). However, VIF values >5.0 may still reflect evidence of multicollinearity. To check for the effects of these higher VIFs, for models with covariates with VIF >5.0, we ran an additional model dropping the variable with the highest VIF and ensured that the remaining selection gradients fell within the 95% confidence intervals from the original model (as in Emel et al., 2017).

We calculated selection gradients (*β*) as the partial regression coefficients simultaneously fitted to all traits in a single multiple regression analysis. We calculated linear selection differentials (*s*') as the covariance between each trait and relative fitness; we assessed significance of differentials through the *p*‐value of Pearson correlation tests of each trait on relative fitness. We report significance levels associated with selection coefficients for each trait × population × site for descriptive purposes, but we did not draw conclusions from these individual *p*‐values; thus, we did not correct them for multiple comparisons (that is, we did not apply Bonferroni or other correction). The number of plants analyzed in each population ($\bar{x}\pm 1\;SE$; 75.7 ± 5.4, *n* = 23 populations) did not allow estimates of nonlinear selection gradients or differentials, or tests of correlational selection using trait × trait interactions. Additionally, we calculated Pearson correlations among floral traits for each population and report the average correlation for each pairwise trait combination (Table S3).

We used ANCOVA to assess whether populations experienced different selective pressures near versus far from crop sunflowers, and whether selection varied among sites or years. We used relative fitness as the response variable. We ran a model for 2010 across sites 1, 2, 3, and 5 (omitting site 4 as described above), and a second model for 2010/2011 which included the two sites (1 and 2) where the experiment was replicated in both years. For total selection, we ran ANCOVA models for each trait separately for 2010 and 2010/2011 for Sites 1 and 2. Models included the fixed factors site, proximity to sunflower crop, and their interactions with the trait. We included population as a random effect. The multiyear model was similar to the 2010 model but included year as an additional fixed factor. For direct selection, the models had the same structure as the models above for total selection but included all nine floral traits, the two seed damage traits, plus plant stem volume as an additional covariate. A significant interaction between trait × proximity would indicate that total or direct selection on traits consistently varied with proximity to crop sunflowers, while other significant interactions involving proximity (trait × site × proximity or trait × site × proximity × year, etc.) would indicate that selection on traits varied with proximity but also depended on geographic or temporal context. We performed analyses with the function *lme* in the R package *nlme* (Pinheiro et al., 2015).

### 2.7 Homogeneity of selection

We hypothesized that variation in selective regimes would differ near versus far from crop sunflowers. Using selection gradients (*β*) and selection differentials (*s*') calculated in the above analyses on individual populations, we compared variances using *F*‐tests ($F=s_{\mathrm{far}}^{2}/s_{\mathrm{near}}^{2}$). *F*‐tests are a measure of overall heterogeneity and may therefore obscure any differences in the direction of selection (positive or negative). To prevent differences between traits in direction of selection from affecting the variance calculations in our *F*‐tests of all traits combined, we first recentered the selection differentials and selection gradients to a mean of zero for each trait across populations. Additionally, we conducted individual *F*‐tests for each trait separately (without recentering). Significantly reduced (or increased) variance of selection differentials or gradients in near relative to the far populations would suggest that natural selection is more (or less) homogenous in closer proximity to the crop species, and would indicate that agriculture is associated with large‐scale spatial alteration of the patterns of natural selection.

### 2.8 Structural equation modeling

We used multigroup structural equation models (SEM) to compare the contribution of mutualists versus antagonists to selection on floral traits near versus far from crop sunflowers. We constructed a plausible a priori model that links floral traits to pollen deposition and damage by seed predators and then links the pollen deposition and damage to plant fitness. We ran models separately for each population‐pair (where a population‐pair contains far versus near populations for each site × year × seed source combination). We included inflorescence traits, disk flower traits, *Isophrictis* sp. damage, *N. helianthi* damage, total number of inflorescences, plant stem volume, and relative fitness as variables in the model. For four population‐pairs, we also included pollen deposition data. As there were relatively few plants per population and nine floral traits, we created two summary variables for floral traits (one for inflorescence traits and one for disk flower traits) by extracting the first principal component from two separate principal components analyses (PCAs) using the *vegan* package in R (R Core Development Team, 2016) for each site‐year combination. To improve interpretability, the sign of trait values was switched as necessary so that all traits were positively correlated with the first principal component; thus, positive coefficients in the paths connecting these composite variables to fitness would represent selection for larger trait values. The importance of components and loadings are included in Table S4. We standardized all traits $\overset{-}{x}$ (mean = 0, SD = 1) prior to analysis, and we log‐transformed traits as needed to improve normality; we relativized whole plant seed production to the mean of the population. We conducted piecewise structural equation modeling including a multigroup analysis on near‐far pairs of populations for the population‐pairs (site × year × seed source combinations) with sufficient data (Sites 1 and 2 in 2010 and 2011) using local estimation in the R package *piecewiseSEM* (Lefcheck, 2016). For each population‐pair, we constructed the model and used the multigroup function to iteratively determine whether the effects of each path vary by treatment (near vs. far from crop sunflowers). We used Shipley's test of d‐separation (Shipley, 2009) to calculate Fisher's *C* statistic to evaluate model goodness‐of‐fit and compared this to a *Χ^2^* distribution to obtain a model‐wide *p*‐value. We used individual plants as the units of observation. We summarized these models by presenting the average standardized path coefficients and indicating whether paths were constrained or not far versus near to crop sunflowers.

## 3 RESULTS

### 3.1 Overall patterns of selection on traits

**FIGURE 3:**
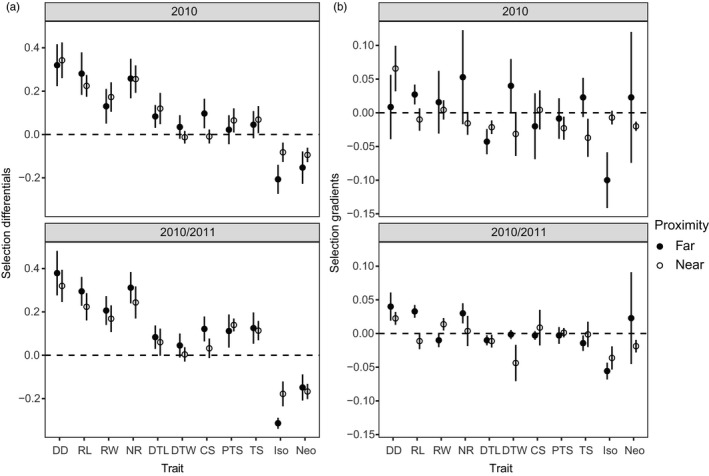
Mean (±1 SE) magnitude of (a) selection differentials and (b) selection gradients for all populations far (filled circle) and near (empty circle) from crop sunflowers from either 2010 (top panels, Sites 1, 2, 3, and 5) or from both 2010 and 2011 (bottom panels, Sites 1 and 2). Values were calculated independently for each population (see Section 2 for details), and then mean values calculated across population values. There are no significance statistics associated with these values calculated within populations, but the values of the coefficients are used in Question 2 in Section 3. Inflorescence traits: CS, corolla lobe size; DD, disk diameter; NR, number of rays; RL, ray length; RW, ray width; floral traits: DTL, distal throat length; DTW, distal throat width; PTS, proximal throat size; TS, corolla tube size; Antagonist traits: ISO, *Isophrictis* attack, NEO, *Neolasioptera* attack

Overall, there was significant total selection (*s*') in 29% of the cases measured (74 out of 253 combinations of 11 traits × 23 populations; Table S5; Figure 3), compared with 5% of cases (13) that could be expected by chance. Total selection was more often significant on inflorescence level traits (54%, 50 out of 92) than on disk flower traits (10%, 11 out of 115) or antagonist traits (33%, 15 out of 46). There was significant total selection on a large percentage of populations on inflorescence level traits, for example, for increased disk diameter (56% of populations), increased number of rays (56%), increased ray length (61%), and increased ray width (43%; one population experienced selection for decreased ray width; Table S5). Fewer populations experienced significant total selection on disk flower traits, for example, for increased corolla lobe size (9% of populations), increased distal throat width (0%), increased distal throat length (9%), increased proximal throat size (17%), and increased corolla tube size (13%). Populations varied in terms of whether they experienced significant total selection on antagonist‐related traits, for example, for lower *Isophrictis* attack (43%) and lower *Neolasioptera* attack (22%).

Selection gradients (*β*) revealed that some of the total selection was due to selection on correlated characters. Significant direct selection was found in 12% of the cases measured (30 out of 242; Table S6; Figure 3), compared with 5% (12) that might be expected by chance. Direct selection was significant in some inflorescence level traits (9% or 8 out of 88 cases) and individual flower level traits (8% or 9 out of 110 cases). Direct selection was significant in 30% (13 out of 44) cases for antagonist traits. There was significant direct selection in a small percentage of populations on inflorescence level traits, for example, for increased disk diameter (18% of populations), increased number of rays (14%), and increased ray width (5%). Very few populations experienced significant direct selection on individual flower traits, for example, for decreased distal throat width (14%), decreased distal throat length (5%), decreased proximal throat size (9%), and decreased corolla tube size (14%). Direct selection on antagonist‐related traits varied, with selection for decreased *Isophrictis* damage in 36% of cases and selection for decreased *Neolasioptera* damage in 14% of cases. Unexpectedly, there was also significant direct selection for increased *Neolasioptera* damage in two cases (9%).

### 3.2 Q1) How does proximity to crop sunflowers affect selection on *H. a. texanus* floral traits?

#### 3.2.1 Total selection across all populations

Proximity to crop sunflowers affected total selection on resistance to antagonists (measured by selection differentials, *s*') in 2010 or 2011, where we interpreted cases where there was a significant trait × proximity effect in ANCOVA as evidence of differential selection near versus far from crop sunflowers. In 2010, total selection on floral traits differed by proximity to crop sunflowers for *Neolasioptera* attack (Figure 3a top panel, Table 1); the overall trait × proximity difference was largely in magnitude rather than direction, as the selection differentials were negative in all but two populations, and the average selection differential was −0.15 far from crop sunflowers and −0.10 near to sunflowers, while there was also a significant trait × proximity × site effect (Table S5). Proximity did not exhibit overall effects on total selection for any of the remaining ten traits analyzed (Table 1). Although there were significant trait × proximity × site effects in four additional traits (disk diameter, ray length, number of rays, and proximal throat size, Table 1), when examining results for individual populations, the average selection differentials were all in the same direction and of similar magnitude (Table S5). For instance, the average selection differential for disk diameter is 0.32 far from crop sunflowers and 0.34 near crop sunflowers, and similar patterns are found for the other traits (Figure 3a top panel, Table S5). These patterns indicate that for these four traits, the proximity effects varied by site but showed no consistent near versus far differences.

**TABLE 1:** Results of analysis of covariance testing for differences in total selection (selection differentials, *s*') due to proximity to sunflower crops and site in 2010, and for Sites 1 and 2 in 2010 and 2011. Reported values are *p*‐values. Separate ANCOVA models were run for each trait to compare selection differentials (total selection) among factors. Data include those for Sites 1, 2, 3, and 5 (see Figure 1). Numerator degrees of freedom (ndf) were the same in all models; denominator degrees of freedom (ddf) varied among models, so the range is provided

Variable	ndf	ddf	DD	RL	RW	NR	DTL	DTW	CS	PTS	TS	ISO	NEO
2010													
Site	3	6	0.999	0.995	0.988	0.992	0.954	0.965	0.968	0.967	0.957	0.914	0.916
Proximity	1	6	0.908	0.893	0.851	0.846	0.993	0.946	0.944	0.947	0.908	0.647	0.650
Site × Proximity	3	6	0.868	0.871	0.866	0.821	0.845	0.778	0.802	0.773	0.809	0.850	0.954
Trait	1	978–1058	**<0.001**	**<0.001**	**<0.001**	**<0.001**	**0.003**	0.933	0.182	0.203	**0.049**	**<0.001**	**<0.001**
Trait × Site	3	978–1058	**<0.001**	**<0.001**	**0.002**	**<0.001**	**0.006**	*0.059*	0.272	**0.017**	**0.032**	**<0.001**	**0.003**
Trait × Proximity	1	978–1058	0.309	0.655	0.470	0.385	0.845	0.778	0.142	0.773	0.809	0.953	**0.024**
Trait × Site × Proximity	3	978–1058	**0.005**	**0.025**	0.815	**0.005**	0.975	0.667	0.235	**0.009**	0.680	*0.065*	**0.042**
2010/2011													
Year	1	7	**0.034**	*0.074*	*0.094*	*0.067*	0.654	0.692	0.689	0.624	0.640	*0.085*	*0.094*
Site	1	7	0.894	0.975	0.990	0.996	0.806	0.920	0.961	0.985	0.991	0.551	0.562
Proximity	1	7	0.894	0.921	0.961	0.976	0.810	0.956	0.963	0.958	0.968	0.822	0.827
Year × Proximity	1	7	0.442	0.478	0.550	0.507	0.383	0.408	0.425	0.414	0.404	0.548	0.559
Site × Proximity	1	7	0.261	0.281	0.333	0.323	0.152	0.273	0.267	0.279	0.266	0.512	0.523
Year × Site × Proximity	1	7	0.548	0.553	0.648	0.732	0.148	0.185	0.180	0.159	0.167	0.277	0.290
Trait	1	1149–1268	**<0.001**	**<0.001**	**<0.001**	**<0.001**	**0.008**	0.452	**0.017**	**<0.001**	**<0.001**	**<0.001**	**<0.001**
Trait × Year	1	1149–1268	0.506	0.999	0.835	0.726	0.644	0.131	*0.072*	*0.083*	0.505	0.306	0.181
Trait × Site	1	1149–1268	**<0.001**	**0.002**	**0.042**	**<0.001**	**<0.001**	*0.074*	0.327	**0.036**	**0.006**	**0.016**	0.309
Trait × Proximity	1	1149–1268	0.521	0.135	0.471	0.901	0.577	0.374	0.121	0.460	0.784	**0.005**	0.737
Trait × Year × Proximity	1	1149–1268	**0.043**	*0.088*	**0.040**	0.266	0.556	0.836	0.499	0.136	0.286	**0.042**	**0.005**
Trait × Site × Proximity	1	1149–1268	**0.024**	**0.011**	**0.002**	0.241	0.624	0.385	0.912	0.246	0.205	**0.022**	**0.024**
Trait × Year × Site × Proximity	1	1149–1268	**<0.001**	**<0.001**	**0.009**	**<0.001**	0.541	0.641	**0.029**	**0.017**	0.911	0.729	0.246

Bold values are significant at *p* < 0.05, italicized values are significant at *p* < 0.10.

In an analysis including 2010 and 2011 data for Sites 1 and 2, we asked whether total selection on floral traits differed by proximity to crop sunflowers while incorporating data from multiple years. Total selection differed by proximity to crop sunflowers overall for *Isophrictis* attack (Table 1). This difference was in magnitude, with average *s*' of −0.31 and −0.18 far from versus near to crop sunflowers, respectively (Figure 3a bottom panel, Table S6). There were also significant trait × proximity × year and trait × proximity × site interactions for *Isophrictis* attack (Table 1). Eight other traits exhibited an effect of proximity when also accounting for either year, site, or both (disk diameter, ray length, ray width, number of rays, corolla lobe size, proximal throat size, *Isophrictis* attack, and *Neolasioptera* attack), but none of these exhibited overall effects of proximity (trait × proximity effects) (Table 1). When examining individual populations, significant selection differentials for these traits also tended to be in the same direction near to and far from crop sunflowers (Table S6).

#### 3.2.2 Direct selection across all populations

We found limited evidence that direct selection (measured by selection gradients, *β*) on floral traits differed by proximity to crop sunflowers (ANCOVA: trait × proximity). In 2010, there was a trend for direct selection on ray length to differ far from versus near to crop sunflowers (*F* = 2.89, *p* = 0.089; Table 2). There was no significant direct selection on ray length in any individual population (Table S6), though the average of selection gradients far from crop sunflowers was *β* = 0.03 and near to crop sunflowers was *β* = −0.01 (Figure 3b top panel). There was also a trend for direct selection on *Neolasioptera* attack to differ far from versus near to crop sunflowers (*F* = 3.11, *p* = 0.078; Table 2). Direct selection on *Neolasioptera* attack was significant in three populations and differed in direction, with selection for decreased attack in one case (from crop sunflowers) and increased attack in two cases (both far from sunflowers) (Figure 3b top panel, Table S6). This resulted in average selection gradients of *β* = 0.02 far from crop sunflowers and *β* = −0.02 near to crop sunflowers (Figure 3b top panel). There was one case with a significant trait × proximity × site effect (disk diameter) (Table 2).

**TABLE 2:** Results of analysis of covariance testing for differences in direct selection (selection gradients, *β*) due to proximity to sunflower crops and site in 2010. A single ANCOVA model was run to compare selection gradients (direct selection) among factors, which includes correlations among traits. Data include those for Sites 1, 2, 3, and 5 (see Figure 1)

Variable	Selection gradients (*β*)			
	ndf	ddf	*F*	*p*
Site	3	6	1.898	0.594
Proximity	1	6	0.000	0.999
Site × Proximity	3	6	1.308	0.727
DD	1	816	11.773	**0.001**
DD × Site	3	816	0.725	0.867
DD × Proximity	1	816	0.228	0.633
DD × Site × Proximity	3	816	10.339	**0.016**
RL	1	816	0.176	0.675
RL × Site	3	816	6.609	0.085
RL × Proximity	1	816	2.889	0.089
RL × Site × Proximity	3	816	1.088	0.780
RW	1	816	0.493	0.483
RW × Site	3	816	0.531	0.912
RW × Proximity	1	816	1.924	0.165
RW × Site × Proximity	3	816	0.517	0.915
NR	1	816	0.730	0.393
NR × Site	3	816	3.378	0.337
NR × Proximity	1	816	0.018	0.892
NR × Site × Proximity	3	816	0.948	0.814
DTL	1	816	6.149	**0.013**
DTL × Site	3	816	0.207	0.976
DTL × Proximity	1	816	0.403	0.525
DTL × Site × Proximity	3	816	0.035	0.998
DTW	1	816	2.438	0.118
DTW × Site	3	816	7.182	*0.066*
DTW × Proximity	1	816	0.007	0.933
DTW × Site × Proximity	3	816	2.238	0.524
CS	1	816	0.270	0.603
CS × Site	3	816	8.914	**0.030**
CS × Proximity	1	816	2.561	0.110
CS × Site × Proximity	3	816	4.353	0.226
PTS	1	816	0.333	0.564
PTS × Site	3	816	1.621	0.655
PTS × Proximity	1	816	0.019	0.892
PTS × Site × Proximity	3	816	0.639	0.888
TS	1	816	1.195	0.274
TS × Site	3	816	0.755	0.860
TS × Proximity	1	816	0.852	0.356
TS × Site × Proximity	3	816	6.026	0.110
Iso	1	816	13.562	**<0.001**
Iso × Site	3	816	5.141	0.162
Iso × Proximity	1	816	1.497	0.221
Iso × Site × Proximity	3	816	2.003	0.572
Neo	1	816	17.945	**<0.001**
Neo × Site	3	816	5.465	0.141
Neo × Proximity	1	816	3.108	*0.078*
Neo × Site × Proximity	3	816	3.712	0.294
Plant vol.	1	816	335.058	**<0.001**
Plant vol. × Site	3	816	17.763	**<0.001**
Plant vol. × Proximity	1	816	0.951	0.329
Plant vol. × Site × Proximity	3	816	16.544	**<0.001**

Bold values are significant at *p* < 0.05, italicized values are significant at *p* < 0.10.

In an analysis including 2010 and 2011 data for Sites 1 and 2, we asked whether direct selection on floral traits differed by proximity while incorporating data from multiple years. There was a trend for direct selection to consistently differ by proximity to crops in one trait (ANCOVA: trait × proximity; Figure 3b bottom panel, Table S8): ray length (*F* = 3.27, *p* = 0.07). Direct selection differed in sign, with selection gradients of 0.03 and −0.02 far from near crop sunflowers respectively (Figure 3b bottom panel). Unlike in 2010, across Sites 1 and 2 in 2010 and 2011, there was no evidence for direct selection differing by proximity in *Neolasioptera* attack (*F* = 0.02, *p* = 0.881, Table S8). Direct selection differed by proximity depending on both site and year in one trait, disk diameter (ANCOVA: trait × proximity × site × year, *F* = 6.65, *p* = 0.010).

### 3.3 Q2) Does homogeneity of selection coefficients and selection gradients for *H. a. texanus* traits differ with proximity to crop sunflowers?

**FIGURE 4:**
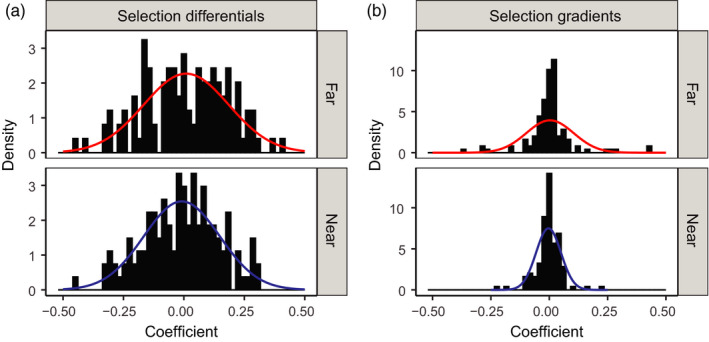
Distributions of standardized selection differentials (a) and standardized selection gradients (b) for all nine traits (see Figure 2) for wild sunflowers grown far from (top) and near to (bottom) crop sunflowers. Normal distributions for each are superimposed over the histograms. Selection differentials represent total selection (direct + indirect selection), whereas selection gradients represent direct selection only. There was a trend for variance of selection differentials to be greater far from crop sunflowers versus near crop sunflowers (*F*_131,143_ = 1.39, *p* = 0.054), and variance of selection gradients was greater near crop sunflowers relative to far from crop sunflowers (*F*_131,143_ = 0.53, *p* < 0.001)

We detected effects of proximity to crop sunflowers on the homogeneity of selection on traits of wild sunflowers. We recentered the selection differentials and selection gradients within a trait to means of zero to account for differences in selection across traits. The variance of recentered selection differentials (total selection on a trait) did not significantly differ far from versus near to crop sunflowers (*F*‐test for homogeneity of variances, ratio of variances [Far/Near] =1.38, *F*_120,131_ = 1.25, *p* = 0.205, Figure 4a), while there was strong evidence that the variance of selection gradients (selection accounting for correlations with other traits) differed, with greater variance far from crop sunflowers relative to near to crop sunflowers (ratio of variances [Far/Near] =3.62, *F*_120,131_ = 3.62, *p* < 0.001; Figure 4b). We also ran *F*‐tests on each trait individually (without recentering). For selection differentials, 8 out of 11 traits (73%) had higher variances far from versus near to crop sunflowers, though the proximity effect was never significant (Table S7). For selection gradients, four traits had significantly higher variances far from versus near to crop sunflowers (ray width, number of rays, *Isophrictis* attack, and *Neolasioptera* attack), and an additional five traits trended in the same direction, for a total of 9 out of 11 traits (82%) with higher variances far from crop sunflowers (Table S7).

### 3.4 Q3) Does selection mediated by mutualists and antagonists differ with proximity to crop sunflowers?

We used multigroup structural equation models to determine whether selection mediated by mutualists (pollinators) and antagonists (seed predators) was the same near versus far from crop sunflowers. In total, we analyzed structural equation models for 11 different population‐pairs, seven of which did not contain pollen deposition data and four of which did. Models for four of the seven population‐pairs without pollen data had good fit, *p*‐values >0.05, and three of the four population‐pairs with pollen data had good fit. Note that in structural equation modeling, the chi‐square tests the null hypothesis that the predictions match the observed data, and therefore, a *p*‐value >0.05 indicates that the model has good fit.

**FIGURE 5:**
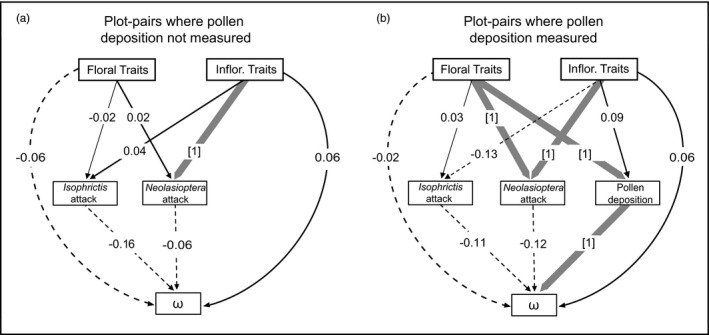
Relationships between wild sunflower traits, activity levels of antagonists and mutualists, and plant fitness as a function of proximity to crop sunflowers. The conceptual diagrams summarize structural equation modeling (SEM) results across seven pairs of populations near and far from the crop with good model support (see Appendix S4 for actual SEM diagrams). Thin solid and thin dashed lines indicate positive and negative paths, respectively, that never differed near versus far; path coefficients shown are the averages across the component SEM models. Thick gray lines indicate paths whose coefficients significantly differed between near and far populations in at least one SEM; numbers in square brackets indicate the number of such SEMs. (a) Summary for averages across four population‐pairs where pollen deposition was not measured. (b) Summary for averages across three population‐pairs where pollen deposition was measured

The effect of antagonists (seed predators) varied near versus far from crop sunflowers. In population‐pairs where pollen deposition was not measured, the relationship between *Neolasioptera* attack and inflorescence traits differed far from versus near to crop sunflowers in one of the four possible cases (Figure 5a). In the population‐pair from 2010 at Site 5 and seed source C, far from crop sunflowers, *Neolasioptera* attack was slightly greater on individuals with smaller inflorescence traits, while near to crop sunflowers *Neolasioptera* attack was slightly greater on individuals with larger inflorescence traits, though neither of these path coefficients were individually significant (*r* = −0.22, *p* = 0.198; *r* = 0.47, *p* = 0.069, respectively) (Appendix S4). No other relevant paths differed far versus near for any of the four population‐pairs without pollen data. When pollen deposition was also measured, a similar effect was found in the population‐pair at Site 1 in 2011 (seed source C), where proximity mediated the relationships between *Neolasioptera* attack and inflorescence size, with greater (though not significant) attack on individuals with smaller inflorescences far from crops (*r* = −0.13, *p* = 0.348) and significantly greater attack on individuals with larger inflorescence traits near to crops (*r* = 0.29, *p* = 0.002). Proximity also mediated the relationship between *Neolasioptera* attack and floral traits, with greater (though not significant) attack on individuals with larger floral traits far from crop sunflowers (*r* = 0.10, *p* = 0.490) and significantly greater attack on individuals with smaller floral traits near to crop sunflowers (*r* = −0.30, *p* = 0.015) (Figure 5b). No paths involving *Isophrictis* attack differed far from versus near to crop sunflowers.

The role of mutualists, as estimated via pollen deposition, also differed far from versus near to crop sunflowers. At Site 1 in 2011 (seed source C), there was greater pollen deposition on individuals with larger floral traits far from crop sunflowers (*r* = 0.40, *p* = 0.003) and no effect of floral trait size near to crop sunflowers (*r* = 0.00, *p* = 0.993) (Figure 5b). In this same population‐pair, the effect of pollen deposition on plant fitness also varied, where far from crops increased pollen deposition resulted in slightly increased relative fitness (*r* = 0.11, *p* = 0.123), but near to crop sunflowers increased pollen deposition resulted in decreased relative fitness (*r* = −0.13, *p* = 0.013).

## 4 DISCUSSION

### 4.1 How does proximity to crop sunflowers affect selection on *H. a. texanus* floral traits?

Global terrestrial land use is dominated by agriculture, which creates homogenized biotic and abiotic environments. However, we know little about how this land use influences evolution by natural selection in plants that occur in agricultural landscapes. We showed that natural selection on heritable floral traits can differ near versus far from crop sunflowers, though detectable differences by proximity were not common, appearing for only three of eleven traits: ray length (a trend) and *Isophrictis* and *Neolasioptera* attack.

### 4.2 Does homogeneity of selection coefficients and selection gradients on *H. a. texanus* floral traits differ with proximity to crop sunflowers?

We found that direct selection was more homogenous near to crop sunflowers (relative to far from). This result was consistent with our expectation that natural selection would be homogenized in *H. a. texanus* populations near crop sunflowers when compared to natural habitats. *Helianthus annuus texanus* populations in agricultural landscapes may experience less diverse selective trajectories near to their crop relatives, which may result in decreased *H. a. texanus* trait diversity near crop sunflowers. This homogenization of selection could especially affect the evolutionary trajectory of wild relatives of *Helianthus*, given the ruderal nature of many taxa and their presence in disturbed habitats such as roadsides and locations adjacent to crops (Rogers et al., 1982).

Alternatively, higher variation in selection far from crop sunflowers could be due to heterogeneity in the environments of the far plots. Individual far plots were planted near semi‐natural habitats that also bordered other non‐sunflower crops, such as sorghum, cotton, corn, rice, or sesame. Therefore, differences in selection among our far plots could be due to variation in the selective environments. Unfortunately, due to the human‐impacted nature of this landscape we were unable to standardize the conditions of the far plots other than to ensure they bordered semi‐natural habitats.

### 4.3 Implications for the geographic mosaic theory of coevolution

Spatial variation in selection in agricultural landscapes may contribute evidence for the geographic mosaic theory (GMT) (Thompson, 2005) in an understudied context. The GMT posits that there is geographic variation in natural selection, reciprocal selection only happens in some locations, and genetic structure constantly changes to alter geographically variable selection. Previous research on the GMT has focused on relatively pristine landscapes (reviewed in Gomulkiewicz et al., 2007). Thompson (2005) posited that human‐generated mosaics would have the same effects on coevolutionary dynamics as natural mosaics but lacked evidence to support this hypothesis. Although we did not address reciprocal selection or genetic structure here, we did find some evidence for one major prediction of the GMT, that the natural selection imposed by one species on another is variable across space. In some ways, our results confirm that natural selection in fragmented agricultural landscapes is similar to natural mosaic landscapes in terms of spatial scale. For example, in this study populations of *H. a. texanus* at the same overall site sometimes differed in selection outcomes near versus far from crop sunflowers over a distance of only ~2.5 km (Tables S5 and S6), patterns seen at similarly small scales in natural landscapes (Anderson et al., 2015; Craig et al., 2007; Gómez et al., 2009; Richardson et al., 2014; Smith et al., 2011). We found that direct selection was more heterogeneous on traits far from the homogenous crop relative to near to the crop, also suggesting that selection outcomes vary across small spatial scales as predicted by the GMT.

### 4.4 Mechanisms for differential selection by proximity to crops

There are several mechanisms that could drive variable selection due to proximity to crops in wild plants. We examined one likely mechanism in our system: alteration of potential biotic agents of selection. Differences in biotic agents near versus far from crop sunflowers could drive differences in selection on plant traits. In previous work, our research group has shown changes in abundance and community structure of mutualist (pollinators) and antagonist (seed predators) putative agents of selection on floral traits in *H. a. texanus* due to crop sunflower proximity (Chamberlain et al., 2013). Specifically, we found that populations of wild sunflowers close to crops supported a greater abundance of pollinators and higher pollinator species turnover across populations, while far from crops wild sunflowers supported more seed predators (Chamberlain et al., 2013). Here, we found that changes in these mutualists and antagonists mediated differences in selection on floral traits at two sites using structural equation modeling (Figure 5). For example, at Site 1 in 2010 (seed source C) attack by the seed predator *Neolasioptera* generated stronger selection on floral traits near to crop sunflowers compared with far from crop sunflowers, consistent with our findings in direct and total selection (Figure 5b, Table 2, Table S8). At that same site in 2011, *Neolasioptera* mediated stronger selection on inflorescence traits near to crop sunflowers, while mutualist pollinators mediated stronger selection on floral traits far from crop sunflowers (Figure 5b). Although SEM by itself cannot determine causation, there is evidence from nature supporting some causal pathways in our models. For example, previous work has demonstrated that seed predators have strong negative effects on plant fitness in *H. a. texanus* (Whitney et al., 2006).

Selection for resistance to antagonists differed far from versus near to crop sunflowers, and the direction of these patterns was reversed for the two seed predators analyzed. Selection for resistance to *Isophrictis* damage was stronger far from crop sunflowers while resistance to *Neolasioptera* damage was stronger near to crop sunflowers (Figure 3). Overall, there was more attack by both types of predators far from crop sunflowers relative to near to crop sunflowers (see also Chamberlain et al., 2013), perhaps due to a concentration effect (fewer congeneric plants to attack, so wild sunflowers were attacked more per capita). The differences in the strength of selection near versus far could be due to different mechanisms. We hypothesize that selection on wild sunflowers imposed by *Isophrictis* damage, which was stronger far from the crop sunflowers, could be due in part to this concentration effect, where near to crops, the decreased overall attack may result in weaker selective pressure overall. Conversely, for *Neolasioptera*, selection for resistance near to crops was stronger, and we hypothesize that this difference could be due to genotypic differences in susceptibility to this pest within the population of wild sunflowers. If individuals vary in their susceptibility to *Neolasioptera* attack, there may still be strong selection against those individuals that are more susceptible even if attack rates are lower.

We found that increased pollen deposition sometimes resulted in significantly lower fitness (Site 1 in 2011, seed source C) (Figure 5b). Although this seems counterintuitive, these plants were not pollen‐limited (Appendix S2) and there is some evidence from other plant species that increased pollen load or pollinator visitation can result in decreased fitness measures (Aizen et al., 2014). For instance, in crop raspberries (*Rubus idaeu*s), increased visitation by invasive *Bombus terrestris* bumblebees resulted in increased damage to styles and the production of fewer drupelets (fruits), also in a system that was not pollen‐limited (Sáez et al., 2014). Other studies have attributed decreased fitness with increased pollen load to interference, thievery, or disease (reviewed in Antonovics, 2005; Young & Young, 1992). Either of these scenarios could be a stronger factor near to crop sunflowers relative to far from crop sunflowers, as crop sunflowers could both attract increased visitation by damaging pollinators or result in interference from heterospecific pollen or disease. Here, we found that increased pollen deposition resulted in lower fitness of wild sunflowers near to crops and slightly higher fitness far from crops (Figure 5b), which could reflect the negative effects of increased pollinators and our previous finding that wild sunflowers near to crops generally supported more pollinators than those far from crops (Chamberlain et al., 2013). Although we did not identify insect visitors or measure damage or disease, sunflower disk florets could be susceptible to these types of damage.

### 4.5 Caveats

We acknowledge a few caveats to our study and how it fits in the broader context of the GMT and effects of agriculture on selection in wild plant species. First, our results may not generalize to systems where a focal wild species grows near an unrelated crop. Crops and unrelated wild plants are less likely to share species interactions, as species interactions are phylogenetically conserved (Gómez et al., 2010). Future studies could manipulate the degree of relatedness between the crop and wild species (for instance, by choosing a more distantly related wild species) to examine whether selection effects differ with relatedness. We also did not have the statistical power in this study to disentangle the effects of agricultural versus wild landscapes in our far plots. Future work could further examine how proximity to not just crop relatives, but any homogenized crop landscape, may affect selection on wild populations. In addition, although some of the focal floral traits in this study experienced selection, different classes of traits may experience different selective consequences due to different agricultural factors. For example, traits related to nutrient acquisition and competition, such as root biomass and growth rate, are likely to experience natural selection due to crop fertilizer runoff and tilling. Floral traits are less likely to respond to these crop management factors and thus may represent a conservative test for the presence of crop proximity effects on natural selection. Finally, populations of wild plants can experience different crop neighbors each year or even within years. Because of this crop rotation, crop effects on natural selection on wild plant traits may be temporally inconsistent, and thus, our findings may not be generalizable.

## 5 CONCLUSIONS

We show that natural selection on mutualist‐ and antagonist‐related traits in a wild plant species (*Helianthus annuus texanus*) was significantly altered by proximity to its crop relative (sunflowers, *H. annuus*). Importantly, total selection on traits in populations of *H. a. texanus* far from their sunflower crop relatives tended to be more heterogeneous compared with populations of *H. a. texanus* near other crops. Furthermore, changes in abundance and community composition of mutualist pollinators and antagonist seed predators mediated differences in selection on floral traits. These results suggest that, despite the common finding that biotic communities are homogenized in agricultural landscapes, there are complex patterns of natural selection on wild species in agricultural landscapes, partly mediated by mutualists and antagonists.

## CONFLICT OF INTEREST

The authors declare no conflict of interest.

## Supporting information

Appendix S1

Appendix S2

Appendix S3

Appendix S4

## Data Availability

Data for this study are available at the Dryad Digital Repository: http://doi.org/10.5061/dryad.bg79cnp9g.
