# Supplementary material for: Proximity to crop relatives determines some patterns of natural selection in a wild sunflower
Source: Evol Appl. 2021 Mar 12;14(5):1328–42. doi: 10.1111/eva.13201 (PMC8127714; doi:10.1111/eva.13201)
Supplement: Supplementary file 1 — Appendix S1 [file EVA-14-1328-s003.docx]

**Supplement S1 – Heritable variation in floral traits of *Helianthus annuus* ssp. *texanus***

Heritability is important because response to selection (R) is a function of selection strength (s) and heritability (*H*^2^) (Lynch & Walsh, 1998) – strongly heritable traits increase response to selection. Thus, if we find that natural selection on floral traits is altered by proximity to agriculture, nonzero heritabilities for these traits would indicate that evolutionary divergence is expected as well. We measured variability in inflorescence and floral traits and calculated narrow-sense heritabilities in *H. a. texanus*.

*Narrow-sense heritability*

As common garden studies cannot rule out maternal effects on phenotypic traits (Roach & Wulff, 1987) we used sire-offspring regression to calculate narrow-sense heritability (Lynch & Walsh, 1998). We measured sire traits and performed controlled crosses in the field in 2011 using a population from seed source B (see main text), then grew the offspring and measured their traits in a greenhouse in 2012, providing a lower bound for the heritability estimate. This approach is superior to measuring heritability only in the greenhouse (Riska, Prout, & Turelli, 1989). We maximized variation among sires by choosing sires that represented the extremes in disk diameter (large and small; a trait to which pollinators previously showed strong responses) (Hill & Thompson, 1977). We bagged inflorescences on randomly chosen dams a few days before hand-pollinating them with sire inflorescences. Sires were only used in one sire × dam combination. In 2012, seeds were germinated in petri dishes, then transferred to round 1 gallon pots with Pro-Mix BX soil mix (BWI, Quakertown, PA) in the greenhouse, with no supplemental light. Plants were fertilized once with 6 g of Osmocote**®** fertilizer (19-6-12 NPK, Scotts-Miracle-Gro**®**, Marysville, OH, USA) when they were approximately 8 cm in height. Plants were watered twice per day for the first month, then watered once every two days afterwards. We used 32 plants and sires and 35 plants as dams, with a mean of 4.6 offspring analyzed per sire (mean ± 1 SE: = 4.6 ± 0.5, range = 1-12).

We calculated narrow-sense heritability of the four inflorescence traits and five disk traits using weighted least-squares sire-offspring regression to account for variation in number of offspring per sire. We calculated weights using the Kempthorne-Tandon derivation from an initial regression, and then reiterated until weighted estimates of the regression coefficient converged (Lynch & Walsh, 1998). Heritability was estimated as 2$\times$ the sire regression coefficient, and a lower bound for heritability was calculated following Riska et al. (1989).

*Results: Narrow-sense heritability*

Narrow-sense heritabilities ranged from 0.15 to 0.64 (Table S1). Five traits displayed heritability values different from zero: disk diameter, ray length, number of rays, distal throat length, and distal throat width. However, four traits showed heritability values not different from zero: ray width, corolla lobe size, proximal throat size, and corolla tube size. These results suggest substantial potential for many *H. a. texanus* floral traits to change in response to natural selection.

Table S1. Results of narrow-sense heritability experiments for four inflorescence, and five floral traits. Narrow-sense values were estimated using sire-offspring regression with sires traits measured in the field, and offspring traits measured in a greenhouse (see Methods section for details). Tests are for heritability estimates significantly different from zero. See Fig. 2 for diagram of floral traits.

| Trait |  | Narrow-sense | |
| --- | --- | --- | --- |
|  |  | *h^2^* | 95% CI |
| Inflorescence traits |  |  |  |
| Disk diameter (DD) |  | 0.47 | (0.318, 0.614) |
| Ray length (RL) |  | 0.28 | (0.001, 0.560) |
| Ray width (RW) |  | 0.25 | (-0.103, 0.602) |
| No. rays (NR) |  | 0.42 | (0.162, 0.681) |
|  |  |  |  |
| Floral traits |  |  |  |
| Distal throat length (DTL) |  | 0.39 | (0.088, 0.696) |
| Distal throat width (DTW) |  | 0.64 | (0.326, 0.955) |
| Corolla lobe size (CS) |  | 0.28 | (-0.018, 0.573) |
| Proximal throat size (PTS) |  | 0.20 | (-0.077, 0.474) |
| Corolla tube size (TS) |  | 0.15 | (-0.161, 0.458) |
|  |  |  |  |

**Literature Cited**

Hill, W. G., & Thompson, R. (1977). Design of experiments to estimate offspring-parent regression using selected parents. *Animal Science*, *24*(2), 163–168.

Lynch, M., & Walsh, B. (1998). *Genetics and analysis of quantitative traits* (Vol. 1). Sinauer Sunderland, MA.

Riska, B., Prout, T., & Turelli, M. (1989). Laboratory estimates of heritabilities and genetic correlations in nature. *Genetics*, *123*(4), 865–871.

Roach, D. A., & Wulff, R. D. (1987). Maternal Effects in Plants. *Annual Review of Ecology and Systematics*, *18*, 209–235. JSTOR. Retrieved from JSTOR.
