# Supplementary material for: Proximity to crop relatives determines some patterns of natural selection in a wild sunflower
Source: Evol Appl. 2021 Mar 12;14(5):1328–42. doi: 10.1111/eva.13201 (PMC8127714; doi:10.1111/eva.13201)
Supplement: Supplementary file 2 — Appendix S2 [file EVA-14-1328-s002.docx]

**Supplement S2 – Pollen limitation in *H. a. texanus* and proximity to crop sunflowers**

We conducted an experiment to test the hypothesis that pollen limitation is greater far from sunflower crops compared to near them. This should result if our hypothesis is true that pollinators should be more abundant near sunflower crops: greater pollinator visitation should lead to decreased pollen limitation (Knight et al., 2005). We randomly selected 40 plants in each of two plots at Site 2, one plot near sunflower crops, and one plot far from sunflower crops. We randomly allocated 20 plants in each plot to the experimental hand-pollination treatment, and 20 to the control, open-pollination treatment. All flowering inflorescences of plants in the hand-pollinated treatment received excess pollen from an entire inflorescence from plants selected at random adjacent to the plot. Control, open-pollinated plants received no extra pollen other than that from pollinator visitation. We measured whole plant fitness on each plant as average seeds per inflorescence (~ 6 inflorescences per plant) × number of inflorescences per plant.

We examined if plant fitness was pollen-limited overall and if the extent of pollen-limitation differed by proximity to sunflower crops using ANOVA. The response variable was whole plant fitness, while the explanatory variables were pollination treatment (hand- vs. open-pollination), proximity to sunflower crops (near vs. far), and their interaction. If the interaction is significant this would suggest that pollen limitation differs by proximity to sunflower crops. We excluded one plant as an extreme outlier. We also determined if plant size (using plant volume) differences could account for differences in plant fitness using ANOVA, separately for each plot.

*Results: Pollen limitation*

There was no pollen limitation either near or far from sun flower crops (ANOVA; *F*_1,74_ = 0.38, *P* = 0.542). In addition, plant fitness did not differ near relative to far from sunflower crops (*F*_1,74_ = 0.16, *P* = 0.688), and there was no interaction between proximity to sunflower crops and the hand-pollination treatment (*F*_1,74_ = 2.26, *P* = 0.137). Plants did not differ in volume in the far plot (ANOVA; *F*_1,35_ = 0.05, *P* = 0.821) or near plot (ANOVA; *F*_1,38_ = 0.32, *P* = 0.575), suggesting that differences in plant size could not account for any differences in fitness between hand- and open-pollination treatments.

**Literature Cited**

Knight, T. M., Steets, J. A., Vamosi, J. C., Mazer, S. J., Burd, M., Campbell, D. R., … Ashman, T.-L. (2005). Pollen limitation of plant reproduction: Pattern and process. *Annu. Rev. Ecol. Evol. Syst.*, *36*, 467–497.
