# Supplementary material for: Proximity to crop relatives determines some patterns of natural selection in a wild sunflower
Source: Evol Appl. 2021 Mar 12;14(5):1328–42. doi: 10.1111/eva.13201 (PMC8127714; doi:10.1111/eva.13201)
Supplement: Supplementary file 4 — Appendix S4 [file EVA-14-1328-s001.docx]

**Supplement S4. Supplementary path analysis results.**


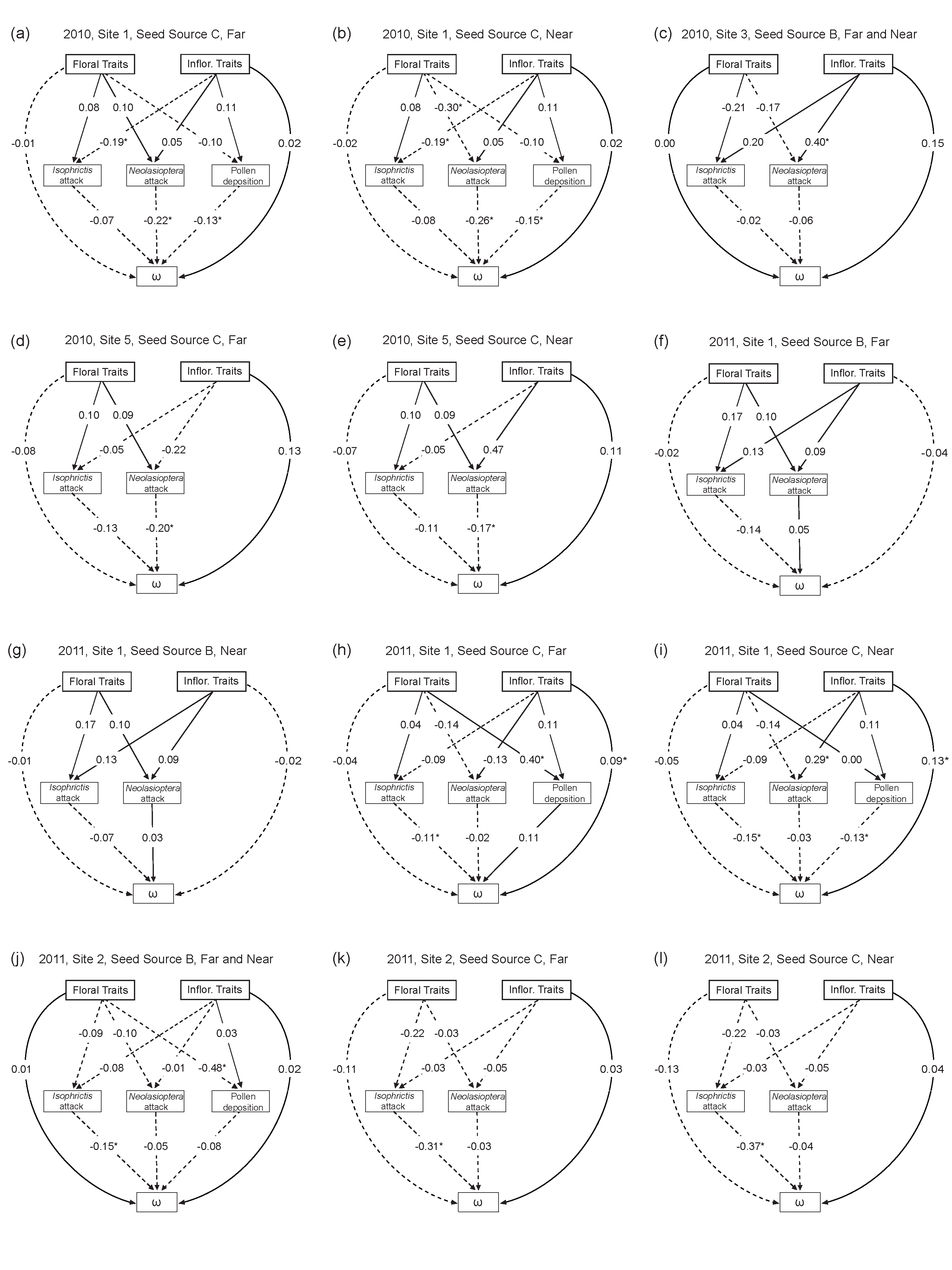
Figure S3. Path analysis results showing standardized coefficients for mutualist versus antagonist mediated selection on floral traits for seven plot-pairs with good model support (*P* > 0.05), 2010 (a-e) and 2011 (f-l) for sites 1 and 2 and seed sources B and C (far versus near, or both). Correlations among exogenous variables, plant number of inflorescences, and plant volume are not shown for clarity. * = Paths determined to be significant (*P* < 0.05). Solid and dashed lines indicate positive and negative paths, respectively. Antagonist seed predators, *Iso*. sp: *Isophrictis* sp.; *N. hel.* = *N. helianthi*. Mutualists, Pollen = pollen deposition. W = *H. a. texanus* relative fitness. Models for plots that were not well supported (P < 0.05).
